# Pharmacokinetics of oseltamivir in infants under the age of 1 year

**DOI:** 10.1186/s40169-016-0118-1

**Published:** 2016-09-05

**Authors:** Rashmi Dixit, Slade Matthews, Gulam Khandaker, Karen Walker, Marino Festa, Robert Booy

**Affiliations:** 1 The Children’s Hospital, Westmead, Sydney, Australia; 2 The University of Sydney, Sydney, Australia

**Keywords:** Oseltamivir, Infants, Influenza, Paediatrics

## Abstract

**Background:**

Oseltamivir is the only antiviral treatment recommended for influenza in young children over the age of 1 year. There is scant data on oseltamivir pharmacokinetics (PK) in infants <1 year. We set out to perform PK measurements in infants who received oseltamivir.

**Methods:**

This study was a prospective, uncontrolled, open label evaluation of the pharmacokinetics of oseltamivir metabolism, safety of oseltamivir, viral clearance in infants <12 months diagnosed with influenza by nasopharyngeal influenza nucleic acid antigen test (NAAT). Blood levels of the prodrug oseltamivir and its active carboxylate were measured prior to a dose of oseltamivir and at 4 time points afterwards, to calculate C_max_ (ng/mL), T_max_ (h), AUC_0−t_ (ng h/mL) and time for AUC (h).

**Results:**

Four children with influenza A received oral oseltamivir, 2.35–3 mg/kg/dose. This dose range produced a target oseltamivir carboxylate plasma concentration in excess of the proposed 12-h target AUC of 3800 ng h/mL, selected from earlier studies to avert resistance. One patient developed GIT adverse event: dry retching.

**Conclusion:**

Oseltamivir was well tolerated at a dose of 2.35–3 mg/kg/dose twice a day in infants under the age of 1 year. In general agreement with earlier data, these doses produced a target oseltamivir carboxylate plasma exposure in excess of the proposed 12-h target exposure of AUC equal to 3800 ng h/mL in two patients. The limited plasma concentration data in the remaining two patients were not inconsistent with the target exposure being reached.

## Background

Infants and young children are particularly prone to influenza morbidity [1–3]. Influenza morbidity in young children and infants ranges from school absenteeism to acute respiratory distress requiring hospitalisation, and can result in death from complications [1]. Oseltamivir is currently the only antiviral treatment recommended in young children, usually for those aged 1–5 years [4–6]. It inhibits the envelope protein neuraminidase, blocking release of viral progeny from infected cells, preventing subsequent entry into uninfected cells [7]. If commenced within 48 h of symptom onset, oseltamivir reduces both duration and complications of influenza [8, 9], although some dispute this [10, 11]. In December 2012, the use of oseltamivir for influenza treatment, but not for prophylaxis, was approved by the FDA for infants as young as 2 weeks, previously having temporary approval for use in infancy during the 2009 pandemic, from April 2009 to June 2010 [12, 13]. Routine use of oseltamivir in infants <1 year of age has been limited by both a lack of pharmacokinetic (PK) data and concern about adverse events [14–17].

The ontogeny of pharmacokinetic functions has potential dosing implications in infants [18, 19]. Oo et al. proposed a dose of 2–3 mg/kg in infants 6–12 months of age, given that renal and hepatic clearance of oseltamivir adjusted for body surface area reach adult levels by 6–9/12 of age [20]. The only known published data regarding oseltamivir pharmacokinetics in infants <1 year old is by Kimberlin et al. [21]. They recommended doses of 3.0 mg/kg twice a day (BID) for infants less than 8 months old, and 3.5 mg/kg BID for infants 9–11 months, based on evidence that these doses achieve an oseltamivir carboxylate 12 h area-under-the-curve (AUC) target of 3800 ng h/mL, and promote less oseltamivir resistance than lower doses [21–24]. The FDA recommends 3 mg/kg BID for infants <1 year of age [25].

During the 2011 influenza season at the Children’s Hospital, Westmead (CHW) in Sydney, Australia, pharmacokinetic data was collected from a series of four infants admitted to intensive care and treated with oseltamivir.

## Methods

### Study population

Infants aged <12 months who warranted treatment with oseltamivir for influenza-like illness were included. The Sydney Children’s Hospitals Network Human Research Ethics Committee provided ethics approval (approval number: HREC/10/CHW/61). All patients or caregivers signed informed consent forms.

### Study design and end points

This study was a prospective, open label evaluation of the pharmacokinetics of oseltamivir metabolism, safety of oseltamivir, viral clearance. The oseltamivir dose prescribed was at the attending clinician’s discretion.

### Pharmacokinetic analysis

Specific recommendations were made for the timing of blood samples to measure levels of oseltamivir and oseltamivir carboxylate. However, to minimize the number of tests and patient discomfort, samples were collected at the same time as clinically required samples, whenever possible. Recommended times of sample collection were within 15 min prior to an oseltamivir dose, 1 h ± 15 min, 2–3 h, 5–7 h and 10–12 h post dose. The blood volume required for plasma level determination was 500 µL. Blood was collected into a sodium fluoride/EDTA collection tube, placed on ice and centrifuged (1500 g at 4 °C for 10 min). Plasma was stored at −70 to −80 °C before despatch to the laboratory (PRA Early Development Services, Inc. Kansas, USA). Oseltamivir and oseltamivir carboxylate concentrations were determined by high-performance liquid chromatography with tandem mass spectrometric detection [18].

We adopted a desirable target exposure value proposed by Kimberlin of an AUC_12_ for oseltamivir carboxylate of 3800 ng h/mL [21]. For computational purposes, concentrations at t = −15 min were taken as concentration at zero time. Non-compartmental analysis was conducted using PKSolver, a published pharmacokinetic analysis Excel plugin [26] to obtain estimates of exposure including AUC_0−t_ and C_max_. A set of 5 time-points from zero to 10 h in the 6th dose cycle was available for two of the four patients while for the other two patients only two time-points were available each, for one patient in the 7th dose-cycle and for the other patient in the 8th dose-cycle. It can be assumed that the patients were at steady-state by this time hence the sparsely sampled data may still give an impression of the exposure to oseltamivir carboxylate in these patients.

### Virological analysis

Each nasopharyngeal swab or aspirate was obtained using a sterile synthetic tip swab, with a plastic or aluminium shaft, and inserted into vials containing sterile viral transport medium. These were collected at treatment initiation and analysed at the Children’s Hospital, Westmead; Sydney, Australia. Influenza was diagnosed and strain type determined using nucleic acid amplification testing (NAAT) by polymerase chain reaction (PCR).

### Safety evaluation

An adverse event was defined as any untoward medical occurrence in a patient which may or may not have a causal relationship with the administered oseltamivir. The following biomarkers were assessed during oseltamivir treatment and compared to pre-treatment levels: serum creatinine, electrolytes, liver transaminases (AST, ALT), alkaline phosphatase, total bilirubin level and full blood count.

## Results

Four children received oral oseltamivir: three at 3 mg/kg twice a day (bd) and one at 2.35 mg/kg bd.

All four patients were infected with influenza A, patients 1–3 were H1N1 and the strain was not documented for the patient 4.

The following table presents the pharmacokinetic parameters for oseltamivir in these four patients (Table 1).

**Table 1:** Patient and pharmacokinetic data for oseltamivir carboxylate in children receiving oral oseltamivir twice a day

Patient number	Age months (m) and days (d)	Sex	Weight (kg)	Dose (mg/kg/dose)	Dose cycle	C_max_ (ng/mL)	T_max_ (h)	AUC_0−t_ (ng h/mL)	Time for AUC (h)	No. plasma samples
1	6 m, 23 d	F	9.05	3	6	772	9.4	6376	0–9.4	5
2	5 m, 13 d	F	9.30	3	6	1960	5.8	18,816	0–10	5
3	11 m, 15 d	F	5.69	3	7	443	3.7	1747	3.7–6	2
4	3 m, 13 d	M	6.43	2.35	8	466	11.5	2156	5–11.5	2

**Fig. 1:**
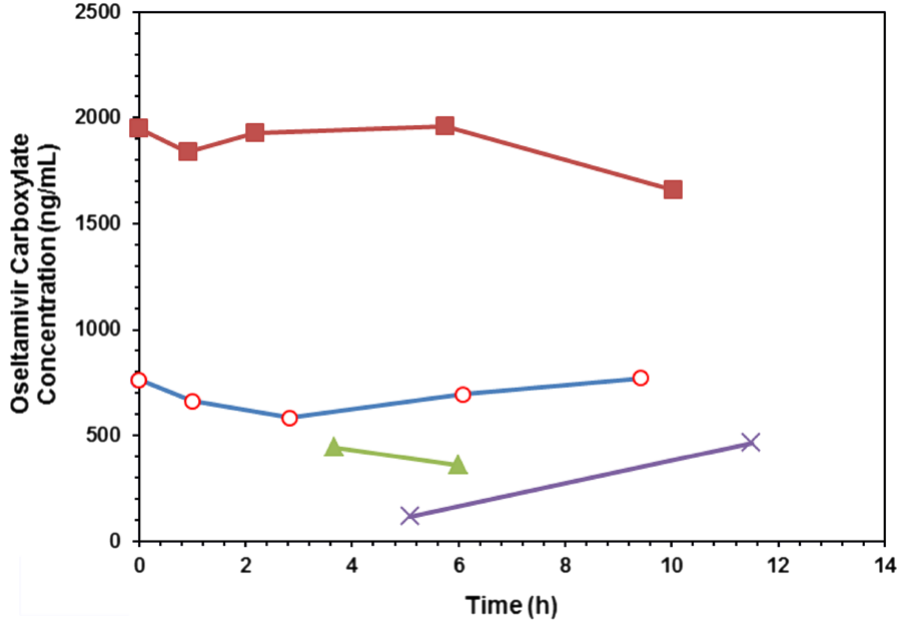
Plasma-time curves for oseltamivir carboxylate in four infants

From the AUC_0−t_ estimates, the first two patients (1 and 2) attained oseltamivir carboxylate plasma concentrations in excess of the proposed 12-h target AUC value for antiviral therapy during the 0 to approximately 10-h period; it can be surmised that a 0–12 h AUC exposure value would also be in excess of the proposed therapeutic target given these AUC_0−t_ values (Fig. 1). The two patients with only two samples per dose cycle were exposed to 1747 and 2156 ng h/mL for periods of 2.3 and 6.5 h, respectively. These levels of exposure are not inconsistent with adequate oseltamivir carboxylate exposure sufficient to provide effective therapy given the proposed target AUC_12_ of 3800 ng h/mL, but further plasma time points would have allowed for confirmation.

All patients recovered from acute influenza during their intensive care unit admissions. One infant suffered an adverse event: self-limited dry retching (Table 2).

**Table 2:** Adverse events (AE) in children receiving oral oseltamivir

Patient number	Age months (m) and days (d)	Comorbidities	Clinical AE	Laboratory changes
1	6 m, 23 d	Tetralogy of Fallot	D1 Dry-retching	Day 1 bloods: raised creatinine (48 mmol/L) and AST (66 mmol/L) Attributed to cardiac condition Normalised after frusemide dose during course of oseltamivir
2	5 m, 13 d	Albright’s osteodystrophe Hypothyroidism Hypoparathyroidism GORD Severe OSA due to epiglottis dystrophy	Nil	Nil
3	11 m, 15 d	*Streptococcus pneumoniae* bacteraemia, pneumonia, meningitis Parainfluenza 3/rhinovirus/enterovirus co-infection Developed HUS	Nil	Baseline bloods normal D2 of oseltamivir: rising creatinine, urea, AST/ALT/GGT All parameters normalised 10 days after first dose Laboratory abnormalities attributed to HUS
4	3 m, 13 d	Exomphthalmos	Nil	Nil

*GORD* gastro-oesophageal reflux disease, *OSA* obstructive sleep apnoea, *HUS* haemolytic uraemic syndrome

## Discussion

Our results are consistent with the proposition that a dose of 2.35–3 mg/kg produced a target oseltamivir carboxylate plasma concentration in excess of the proposed 12-h target AUC of 3800 ng h/mL.

Oseltamivir is well absorbed from an early age. Animal studies demonstrate a rapid increase of the transport protein at birth, and a widespread distribution for oseltamivir including good penetration of lung tissue, the middle ear and the nasal mucosa [27]. It is metabolized to the active metabolite oseltamivir carboxylate by the liver carboxylesterase HCE1 [19, 27]. Production of HCE1 is lower in foetuses than in infants <1 year of age, who in turn have lower gene transcription than children 1–10 years [18]. However, much inter-individual variability exists, particularly in the younger age groups. Young children have greater proportionate extracellular fluid and thus a greater volume of distribution (VD) of oseltamivir carboxylate, resulting in a lower circulating plasma concentration compared to older children and adults [27]. Oseltamivir carboxylate is not extensively protein bound and, thus, immaturity of plasma protein levels does not impact on VD [27]. Animal studies indicate good penetration of oseltamivir carboxylate into respiratory tissues [19]. Oseltamivir has been linked to neuropsychiatric side effects in children and young adults, especially in Japan, although it is unclear whether the encephalopathy was induced by influenza or by its treatment [28, 29]. Both rat and human foetus studies showed certain central nervous system (CNS) efflux pumps to be in low numbers at birth and increase with age, whilst others are present from the second trimester [19]. There was, however, no accumulation of oseltamivir carboxylate in the brains of healthy rats. Oseltamivir is filtered and actively excreted from the renal tubules using OAT transporter proteins [27]. Clearance function of these proteins is low at birth and increases over the first year of life, which may lead to reduced oseltamivir clearance in neonates [27]. Oo et al. demonstrated that oseltamivir carboxylate clearance adjusted for body surface area (BSA) reached adult levels by 6–9 months of age, whilst a higher BSA-to weight ratio in those 1–2 years resulted in higher clearance and consequently lower peak plasma concentration (C_max_), time to reach C_max_ (T_max_) and AUC compared to those 3–5 years [20]. Kimberlin et al. achieved their target AUC with doses of 3 mg/kg in those up to 9 months of age whereas those 9–11 months of age required a higher dose of 3.5 mg/kg, due to greater oseltamivir carboxylate clearance over the first year of life [21]. Thus, oseltamivir clearance may peak around 12 months, and then reduce after 3 years.

There was one GIT side effect (dry-retching) from oseltamivir in our cohort. Laboratory anomalies were attributable to comorbidities. Likewise, a dose of 3–3.5 mg/kg of oseltamivir was well tolerated in 87 infants with no premature drug discontinuation [21]. Of eight adverse events (AE) deemed related to oseltamivir (9.1 %), five were emesis, two developed a rash and one developed a serious AE: cutaneous hypersensitivity. There were no CNS AE. In another trial, 11 infants who received a rather high median dose of 5.5 mg/kg/dose of oseltamivir suffered no serious adverse events, and all completed the course [30]. Two developed a rash, two gastrointestinal side effects and three had transiently raised liver transaminases that normalised within 2 weeks of completing therapy. In a report of 35 patients <1 year of age who received oseltamivir, no AE occurred and no effect on liver function was detected [31]. In a report of 5 premature infants, mean gestational age 31 weeks, who received oseltamivir at 2–3 mg/kg/dose, there were no treatment related AE [32].

## Conclusion

Oseltamivir was well tolerated at a dose of 2.35–3 mg/kg/dose twice a day in infants under the age of 1 year. These doses were confirmed to produce a target oseltamivir carboxylate plasma exposure in excess of the proposed 12-h target exposure of AUC 3800 ng h/mL in two patients and the limited plasma concentration data in the remaining two patients were not inconsistent with the target exposure being reached.
